# Molecular Prevalence and Genetic Characterization of Bovine Tick-Borne Protozoa in Thai and Imported Beef Cattle in Thai-Myanmar Border and Infesting Ticks from Kanchanaburi Province, Thailand

**DOI:** 10.3390/pathogens15040365

**Published:** 2026-03-30

**Authors:** Prottoy Bhadury, Thom Do, Narisorn Pilean, Wissanuwat Chimnoi, Ketsarin Kamyingkird, Xuenan Xuan, Tawin Inpankaew

**Affiliations:** 1 Department of Parasitology, Faculty of Veterinary Medicine, Kasetsart University, Bangkok 10900, Thailand; prottoy.b@ku.th (P.B.); fvetwic@ku.ac.th (W.C.); ketsarin.ka@ku.th (K.K.); 2 National Research Center for Protozoan Diseases, Obihiro University of Agriculture and Veterinary Medicine, Obihiro 080-8555, Japan; thanhthomdo@gmail.com (T.D.); ggen@g.ecc.u-tokyo.ac.jp (X.X.); 3 Department of Livestock Development, Sa Kaeo Animal Quarantine Station, Sa Kaeo 27180, Thailand; narisornpiper@gmail.com; 4 Research Center for Asian Infectious Diseases, The Institute of Medical Science, The University of Tokyo, Tokyo 108-8639, Japan

**Keywords:** piroplasm infection, cross-border cattle, tick-borne protozoa, molecular epidemiology, Thailand

## Abstract

Tick-borne protozoa (TBP), including *Babesia* spp. and *Theileria* spp., cause substantial health and productivity losses in cattle. In Thailand, most epidemiological studies have focused on dairy herds, while beef cattle remain underinvestigated. This study assessed TBP infections in beef cattle and their ticks at the Thai–Myanmar border. Blood samples were collected from 158 beef cattle, including local animals from Thong Pha Phum and Sangkhlaburi districts and cross-border cattle imported from Myanmar. Engorged ticks, predominantly *Rhipicephalus microplus*, were removed and identified morphologically. DNA was extracted from blood and tick samples, and PCR assays were performed to detect *Babesia* and *Theileria* species. Overall, 51.3% of cattle were positive for *Babesia* or *Theileria* DNA. *Babesia bigemina* (10.8%) and *Babesia bovis* (8.2%) were the most frequently detected species. Infection prevalence was higher in local cattle from Thong Pha Phum (56.0%) and Sangkhlaburi (54.6%) than in cross-border cattle (39.5%). In contrast, ticks collected from cross-border cattle showed a significantly higher prevalence of TBP DNA (40.0%) compared with ticks from Thong Pha Phum (12.8%) and Sangkhlaburi (8.7%). These findings provide important epidemiological evidence of TBP circulation at the Thai–Myanmar border and highlight the influence of cattle movement and tick exposure on pathogen distribution and spread in this region.

## 1. Introduction

Tick-borne diseases (TBD) pose a significant challenge to livestock productivity in tropical and subtropical regions, where favorable environmental conditions facilitate both tick growth and pathogen transmission. In Thailand, the widespread presence of vector species such as *Rhipicephalus microplus* and *Haemaphysalis bispinosa* maintains the transmission of tick-borne protozoa (TBP) among ruminant populations. Protozoa of the genera *Babesia* and *Theileria* are responsible for significant economic losses through reduced growth, milk production, reproductive efficiency, and occasional mortality during severe infection [1].

Bovine babesiosis and theileriosis are among the most prevalent TBD in Southeast Asia. *Babesia bovis* and *Babesia bigemina* typically cause pyrexia, hemolytic anemia, and jaundice [2,3], while tropical theileriosis, caused by *Theileria annulata*, leads to the invasion of bovine lymphocytes and erythrocytes, resulting in progressive anemia, lymphadenopathy, and emaciation [4]. Co-infection with these protozoa is common and may intensify clinical manifestations while complicating diagnosis [5].

The beef cattle sector in Thailand plays a vital role in national agriculture, supporting rural livelihoods and contributing to the domestic meat supply. Common breeds include Thai native, Brahman, Charolais, and Simmental, with the Thai native being particularly well adapted to the hot, humid climate and local feed resources. However, these environmental conditions also promote tick proliferation, facilitating disease transmission. Moreover, seasonal climatic variation further stimulates vector activity and infection dynamics, with several studies correlating husbandry and environmental factors to spatial and temporal patterns in TBD prevalence [6]. Molecular investigations have revealed the circulation of *B. bovis*, *B. bigemina*, and *T. annulata* in several provinces, indicating that multiple TBPs are endemic in the region [7,8].

Despite several molecular studies on TBD in Thailand, significant knowledge gaps still exist. Most previous research has focused on dairy cattle or small ruminants, leaving the epidemiology of beef cattle largely unexamined. Geographic coverage has also been limited, with few surveys from key beef-producing regions. Additionally, many studies relied solely on single-gene PCR detection without detailed genetic analysis, which restricts understanding of strain diversity and distribution. Therefore, a targeted molecular study on beef cattle is necessary to clarify the prevalence, genetic diversity, and epidemiological patterns of *Babesia* and *Theileria* spp. in Thailand.

To date, no molecular studies have characterized TBP in cross-border imported beef cattle along the Thai-Myanmar border.

The present study aimed to determine the molecular prevalence of major TBP in beef cattle along the Thai-Myanmar border using PCR-based assays, identify circulating *Babesia* and *Theileria* species, and characterize the detected species by partial gene sequence analysis and comparison with reference strains.

## 2. Materials and Methods

### 2.1. Study Area and Sample Collection

The minimum sample size required for this cross-sectional study was calculated using the formula described by Michael Thrusfield for prevalent studies:

$${n=Z}^{2}P_{exp}(1-P_{exp})/d^{2}$$

where *n* represents the required sample size, *Z* corresponds to the desired confidence level (1.96 for 95% confidence), *P_exp_* is the expected prevalence, and *d* is the desired absolute precision [9]. Based on previously reported molecular detection rates of bovine TBP in Southeast Asia, the expected prevalence (*P_exp_*) was assumed to be 10% [10]. Using a 95% confidence level and an absolute precision of 5%, the minimum required sample size was calculated to be 139 animals. Therefore, the total number of cattle samples in this study was increased (*n* = 158) to ensure sufficient statistical power and to account for potential sample attrition.

**Figure 1:**
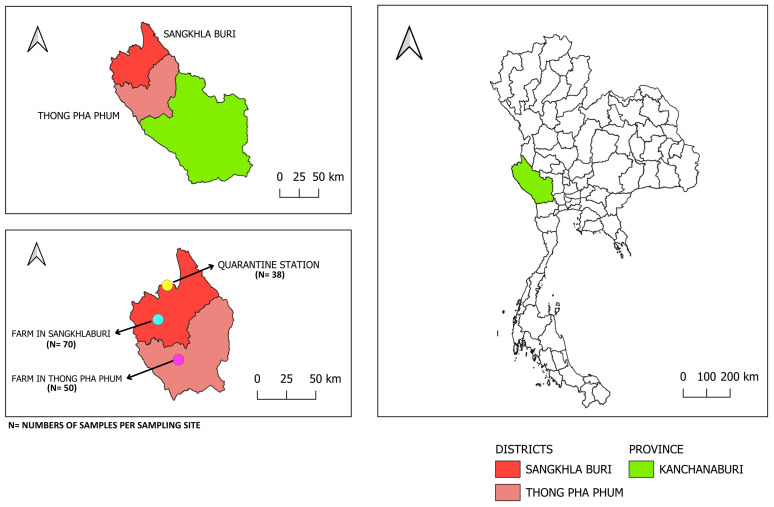
Geographic location of the study area in Kanchanaburi Province, western Thailand. The map shows the location of Sangkhlaburi and Thong Pha Phum districts along the Thai-Myanmar border, including cattle farms and the quarantine station from which samples were collected.

The study was conducted between December 2022 and January 2023 in Kanchanaburi province, western Thailand, a location along the Thai-Myanmar border. A total of 158 blood samples were collected from beef cattle representing two districts, including 50 from Thong Pha Phum (14.840545° N, 98.492435° E) and 70 from Sangkhlaburi (15.150031° N, 98.450269° E), and from 38 cross-border cattle (CBC) group sampled at Kanchanaburi quarantine station (15.256216° N, 98.419401° E; Sangkhlaburi) in Kanchanaburi. The geographic locations of the sampling sites are shown in Figure 1. The CBC group comprised imported cattle originating from Myanmar that were held under quarantine for health inspection before further movement.

Animals were selected using a non-probabilistic convenience sampling method, based on farm accessibility, cattle availability, and farmer consent during the sampling period. The sampled populated included both local and crossbred beef cattle raised under semi-intensive management systems. Apparently healthy cattle present on farms during the sampling period were included in the study, whereas cattle that had recently received antiparasitic treatment or were clinically unfit for sampling were excluded.

Approximately 2–3 mL of blood was collected aseptically from the jugular vein into EDTA-coated vacutainer tubes using sterile needles. Samples were transported on ice to the parasitology laboratory, Faculty of Veterinary Medicine, Kasetsart University, within 24 h and stored at 4 °C until DNA extraction. All animal handling procedures followed the ethical standards approved by the Kasetsart University Animal Care and Use Committee (Approval No: ACKU66-VET-024), ensuring animal welfare and biosafety protocols.

### 2.2. Tick Collection and Identification

Ticks infesting the examined cattle were carefully removed using sterile forceps and stored in 70% ethanol. Tick collection was conducted concurrently with cattle blood sampling on the same date to ensure consistency between host and vector sampling. Morphological identification of ticks was performed under a stereomicroscope following standard taxonomic keys [11]. For molecular examination, a subset of ticks collected from cattle (47 cattle from Thong Pha Phum, 46 from Sangkhlaburi, and 20 from the CBC group) was selected (total *n* = 113). All collected ticks were identified to species level and classified by developmental stage. All of the collected ticks were adult. Each tick was rinsed with distilled water, air-dried, and dissected using sterile scalpels. The specimens were processed individually to avoid cross-contamination. These ticks were used to evaluate potential vector carriage of the detected protozoan species.

### 2.3. DNA Extraction

Genomic DNA was extracted from 200 µL of each EDTA-blood sample using the Genomic Mini Kit (Geneaid Biotech Ltd., New Taipei City, Taiwan) according to the manufacturer’s protocol. For tick samples, DNA was extracted using the NucleoSpin^®^ Tissue Kit (Macherey-Nagel, Düren, Germany) according to the manufacturer’s protocol. The final elution volume for both blood and tick DNA was 100 µL, and DNA samples were stored at −20 °C until analysis.

### 2.4. Molecular Detection of Haemoprotozoa

Molecular detection of tick-borne protozoan infections was carried out using a two-step PCR-based diagnostic workflow, consisting of (i) initial screening to detect *Babesia* or *Theileria* DNA, followed by (ii) species-specific conventional PCR (cPCR) or nested PCR (nPCR) assays for identification of individual protozoan species.

All molecular analyses were conducted on DNA extracted from both cattle blood and tick samples collected from the same animals to evaluate the circulation of protozoa between hosts and vectors.

#### 2.4.1. Initial Screening to Detect *Babesia* or *Theileria* spp.

All extracted DNA samples from blood and ticks were initially screened using cPCR targeting a conserved region of the 18S rRNA gene of *Babesia* and *Theileria* spp. to identify piroplasm-positive samples for subsequent species-level analysis. PCR reactions were prepared in a final volume of 25 µL using a commercial 2× PCR Master Mix (Thermo Fisher Scientific, Waltham, MA, USA), species-specific primers, and template DNA. Amplifications were conducted in a Bio-Rad thermal cycler (Bio-Rad, Hercules, CA, USA) using optimized cycling conditions described in Table 1. Positive and negative controls were included in each run, and amplified products were visualized by 1.5% agarose gel electrophoresis.

#### 2.4.2. Species-Specific Detection by cPCR and nPCR

Species-level identification was carried out using cPCR assays for *B. ovata*, *B. mymensingh*, and *T. orientalis*, and nPCR assays for *B. bovis*, *B. bigemina*, *T. parva*, and *T. annulata*. nPCR was employed to enhance detection sensitivity for targets expected to occur at low parasitemia levels. Primer sets, target genes, amplicon sizes, and cycling parameters for both outer and inner reactions are provided in Table 1. The same amplification protocols were applied to DNA extracted from tick samples to allow direct comparison with blood-derived DNA from corresponding cattle groups.

### 2.5. Hematological Analysis

Packed cell volume (PCV) was determined using the microhematocrit method. Briefly, EDTA blood was transferred into microhematocrit capillary tubes, sealed, centrifuged in a microhematocrit centrifuge, and PCV (%) was read using a microhematocrit reader. The procedure followed standard microhematocrit recommendations described in the hematocrit/PCV methodology literature [17]. PCV values were compared between infected and non-infected animals.

### 2.6. Statistical Analysis

Statistical analyses were performed using Stata version 15 (StataCorp, 4905 lakeway Drive, College Station, TX, USA). Prevalence was calculated as the proportion of PCR-positive samples. Associations between TBP infection and selected epidemiological factors were evaluated using the chi-square test. Multivariable logistic regression analysis was performed to identify independent risk factors. Differences in PCV values were assessed using appropriate parametric tests, with statistical significance set at *p* < 0.05.

## 3. Results

A total of 158 cattle from two different districts and the CBC group were examined for *Babesia* or *Theileria* infections. Simultaneously, ticks collected from the same area of animals in Kanchanaburi Province were morphologically identified and screened molecularly to assess the circulation of tick-borne protozoa between hosts and vectors.

### 3.1. Molecular Prevalence and Geographic Distribution

PCR screening of the *18S rRNA* gene showed that 51.3% (95% CI: 43.4–59.0) were positive for Babesia/Theileria DNA (Table 2). Among the species detected, *B. bigemina* (17/158; 10.8%) and *B. bovis* (13/158; 8.2%) were the most prevalent, followed by *T. orientalis* (9/158; 5.7%) and *B. ovata* (7/158; 4.4%). Interestingly, *B. bigemina* showed the highest prevalence, suggesting widespread subclinical infection in the study region.

The infection was highest in Thong Pha Phum (56%; 95% CI: 42.2–69.8), followed by Sangkhlaburi (54.29%; 95% CI: 42.6–65.9), while the lowest rate was detected in the CBC group (39.5%; 95% CI: 23.9–55.0). The chi-square analysis indicated a significant difference in the overall infection rates among three areas (*p* = 0.015), suggesting geographic influence on pathogen circulation.

Cattle in Thong Pha Phum district showed co-circulation of *B. bovis* (14%), *B. bigemina* (12%), and *T. orientalis* (4%), with no detection of *B. ovata*. This pattern suggests endemicity of multiple *Babesia* species within the same population.

In Sangkhlaburi district, all four protozoan species were detected, with *B. bigemina* again predominating (14.3%), followed by *B. bovis*, *B. ovata*, and *T. orientalis*. The concurrent presence of all species in this district indicates greater ecological diversity of tick-borne protozoa and a potential risk of mixed infections.

Conversely, the CBC group presented a distinct infection profile, characterized by the complete absence of *B. bovis* but higher prevalence of *B. ovata* (13.2%) and *T. orientalis* (15.8%) compared to the other districts. *B. bigemina* was detected at a low level (2.6%). The predominance of *B. ovata* and *T. orientalis* in this group represents the cross-border cattle population. It suggested the possible introduction of these species through transboundary cattle movement and subsequent adaptation to local tick vectors along the Thai-Myanmar interface.

Co-infections were identified in 11 out of 158 examined cattle, resulting in an overall prevalence of 6.96% (11/158, 95% CI: 3.93–12.04). The most common co-infection was the simultaneous detection of *B. bovis* and *B. bigemina*, identified in 6 cattle (3.80%; 95% CI: 1.75–8.04). Co-infection with *B. ovata* and *T. orientalis* was observed in 3 cattle (1.90%; 95% CI: 0.65–5.44). In addition, mixed infections involving *B. bigemina* with *T. orientalis* and *B. bovis* with *B. ovata* were each detected in one animal (0.63%; 95% CI: 0.11–3.50). Overall, these findings indicate that although more than one protozoan co-infection occurred at a relatively low frequency, multiple hemoprotozoan parasites are concurrently circulating within the studied cattle population.

Collectively, spatial comparison revealed that *B. bovis* and *B. bigemina* infections were significantly more prevalent in local Thai cattle populations (Thong Pha Phum and Sangkhlaburi districts), while *B. ovata* and *T. orientalis* infection were strongly associated with the CBC group. These findings emphasized the role of cattle movement and ecological variation in shaping protozoan diversity and transmission dynamics across the Thai-Myanmar border region.

### 3.2. Multivariable Logistic Regression: Association Between Risk Factors and Species-Specific Infection

Host factors with PCR positivity of TBP are summarized in Table 3. Overall prevalence refers to cattle positive for at least one pathogen species.

For overall PCR positivity, prevalence showed only modest variation across host factors categories. Across age categories, overall positivity was comparable, with the highest proportion observed in cattle > 5 years and the lowest in cattle ≤ 2 years. By breed, crossbred cattle showed higher overall positivity than local cattle. By sex, overall positivity was similar between females and males. By ectoparasite status, at sampling, overall positivity was higher among cattle recorded as ectoparasite positive than those recorded as ectoparasite negative.

A multivariable logistic regression analysis was performed to evaluate associations between selected risk factors and species-specific TBP infections. Among the variables examined (sex, breed, age, presence of ectoparasites), statistically significant associations were observed for *B. bigemina*, *B. ovata*, *T. orientalis*, while *B. bovis* showed no marked independent associations.

For *B. bigemina*, sex and ectoparasite status were significantly associated with infection. Female cattle had almost four times higher odds of infection compared with males (odds ratio (OR) = 3.81; *p* = 0.017). In addition, cattle classified as free of ectoparasites at the time of sampling showed increased odds of infection compared with infested cattle (OR = 7.03; *p* = 0.023).

For *B. ovata* and *T. orientalis*, ectoparasite status was also significantly associated with infection. In both cases, cattle without detectable ectoparasites exhibited higher odds of infection (*B. ovata:* OR = 0.05; *p* = 0.036; *T. orientalis*: OR = 0.13; *p* = 0.024). However, the number of positive cases for these species was limited.

No statistically significant association was detected between any evaluated risk factor and *B. bovis* infection. However, a non-significant trend toward higher odds of infection was observed in cattle with ectoparasite infestation (OR = 5.30; *p* = 0.136). Across all parasite species, breed, and age categories did not show consistent or significant associations with infection status. Overall, the results suggest species-specific and study-dependent associations between host factors and infection, which should be interpreted cautiously given the limited number of positive cases for some TBP.

### 3.3. PCV Comparison Between Infected and Non-Infected Cattle

An independent samples *t*-test was conducted to evaluate the effect of *Babesia* or *Theileria* infection on the PCV of cattle. The mean PCV of non-infected animals was 36.21 ± 4.77, whereas infected cattle exhibited a lower mean PCV of 34.60 ± 5.08. The difference between the two groups was statistically significant (*p* = 0.042) (Table 4). This indicates that animals infected with Babesia or *Theileria* had significantly reduced hematocrit values compared to uninfected cattle. The observed decrease in PCV among infected individuals suggests the presence of mild to moderate anemia associated with haemoprotozoan infection, consistent with the pathogenic effects of these protozoa on red blood cell integrity.

### 3.4. Molecular Detections of Tick-Borne Protozoa in Cattle-Associated Ticks

All ticks collected from infested cattle were morphologically identified as *R. microplus* based on standard taxonomic keys. Among the 158 examined cattle, 113 animals (71.5%) carried *R. microplus*. Molecular screening of ticks from these animals revealed notable differences in protozoa detection across study areas. Overall, *Babesia* or *Theileria* positivity was highest in ticks collected from CBC group (40%), significantly exceeding the levels detected in Thong Pha Phum (12.8%) and Sangkhlaburi (8.7%) (*p* = 0.005), as mentioned in Table 5. Species-specific analyses revealed low detection rates of *B. bovis* and *B. bigemina*, with no significant differences between areas. Moreover, no co-infection among protozoa was detected in studied population. Notably, PCR assays targeting *B. ovata* and *T. orientalis* yielded no positive detections in any tick sample, indicating these species were circulating in cattle but were not identified in their infesting ticks at the time of sampling. Although individual *Babesia* species occurred at low frequencies, the substantially higher overall protozoan positivity in ticks from the CBC group indicates that transboundary movement may introduce infected ticks and influence local patterns of parasite circulation.

### 3.5. Molecular Identification and Sequence Homology Analysis of Babesia and Theileria Species in Blood and Tick Samples

Sequence analysis of selected PCR-positive blood and tick samples confirmed the molecular identity of detected haemoprotozoa. In blood samples, the *B. bovis* SBP-4 gene showed 98.1% identity to reference AB772320.1; *B. bigemina* RAP-1a, 98.3% to MK481015.1; *B. ovata* AMA-1, 92.49% to AB703297.1; *T. orientalis* MPSP, 100% to LC438487.1. In ticks, *B. bovis* SBP-4 matched AB772320.1 at 98.1%, and *B. bigemina* RAP-1a matched MK481015.1 at 98.79%. No other *Babesia* or *Theileria* species were detected (Table 6).

## 4. Discussion

This study provides a comprehensive molecular assessment of bovine TBP along the Thai-Myanmar border, integrating host and vector data to delineate infection dynamics in beef cattle and associated ticks. The overall molecular prevalence of *Babesia* or *Theileria* spp. infections (51.3%) detected in beef cattle suggest that haemoprotozoan infections are endemic in Kanchanaburi province. This prevalence is comparable to previous molecular studies conducted in Thailand that reported detection rates ranging from approximately 55% to over 65%, depending on host types, geographic region, and diagnostic targets [5,7,8]. However, marked regional variation has been reported. Northern provinces such as Lampang have shown markedly higher detection rates (63.10%), whereas moderate prevalence has been observed in central regions like Nakhon Pathom (32.86%), and comparatively lower rates have been reported in Kanchanaburi (6.67%) [18]. These differences are likely attributable to variations in cattle density, tick vector abundance, ecological conditions, and management practices.

In this study, *B. bigemina* was the most frequently detected protozoan species and showed significant associations with sex and the presence of ectoparasites in multivariable analysis, highlighting the importance of tick exposure and management-related factors in infection risk. Similar patterns have been reported in South Asia. For example, a molecular survey from Tripura, India, reported a *B. bigemina* prevalence of 4.69% in tick-infested cattle, with infection risk linked to cumulative tick exposure and sex-related management practices [19]. Although the prevalence reported from Tripura was lower than that observed in the present study, both investigations support the role of *B. bigemina* as a persistent and epidemiologically important protozoan in cattle. Consistent findings have also been reported from China, where molecular studies have documented low to moderate *B. bigemina* prevalence (2–6%), particularly in tick-endemic regions [14]. Collectively, these studies indicate that *B. bigemina* is widely distributed across Asian cattle populations, albeit with heterogeneous prevalence influenced by ecological and management factors.

In contrast, *B. bovis* did not show significant associations with evaluated factors in the present analysis, despite its moderate prevalence. This pattern is consistent with previous molecular studies from Thailand, where *B. bigemina* frequently predominates over *B. bovis* in beef cattle, likely due to its efficient transmission by *R. microplus* and its ability to persist as a carrier infection [7,8]. These findings suggest that local ecological conditions and vector dynamics may play a more important role in shaping *B. bovis* transmission than host demographic factors alone.

*B. ovata* was detected at a lower prevalence (4.4%) in the present study but showed a significant association with the presence of ectoparasites, emphasizing the central role of ticks in their transmission. Recent epidemiological data on *B. ovata* are limited. Earlier reports documented prevalence rates of approximately 2.8% in Japan and 2.5% in Thailand [13]. To date, no published studies have specifically examined *B. ovata* infection in cross-border cattle populations in Thailand. The detection of *B. ovata* in both the local and CBC groups in the present study suggests that animal movement along the Thai-Myanmar border may contribute to its introduction and maintenance within this region.

*T. orientalis* was detected at a moderate prevalence, with limited associations with host demographic factors apart from ectoparasite status. Across Asia, reported prevalence of *T. orientalis* varies widely, ranging 0% to 27% in studies from India and East Asia [14,20] to substantially higher prevalence in Malaysia, where nearly half of the examined cattle were infected (49.76%) [21]. These differences suggest that *T. orientalis* transmission is strongly influenced by local vector ecology and environmental conditions rather than following a uniform regional pattern.

To date, published data on TBD in cross-border imported cattle entering Thailand are extremely limited. The present study, therefore, provides the first molecular evidence describing the prevalence of *Babesia* and *Theileria* spp. in imported cattle along the Thai-Myanmar border. The detection of *B. ovata* and *T. orientalis* in this population, together with prevalence patterns distinct from those observed in local cattle, suggests that CBC movement may contribute to the introduction or redistribution of specific piroplasm species. These findings highlight the need for targeted surveillance and molecular screening of imported cattle to mitigate the risk of pathogen introduction and establishment in border areas.

Interestingly, while co-infections were detected in cattle, no co-infections were identified in the examined tick samples collected during the same sampling period. This discrepancy may suggest that cattle acquire different pathogens through repeated exposure to infected ticks over time rather than through simultaneous transmission from a single tick. As cattle can remain persistently infected and act as carrier hosts, successive tick infestations may gradually result in the accumulation of different hemoprotozoan species within the sample cattle. In contrast, the absence of co-infection in ticks may be associated with limited pathogen acquisition during individual feeding events or biological constraints affecting simultaneous pathogen maintenance within the vector. Together, these findings emphasize the epidemiology of host vector interactions influencing the transmission of bovine tick borne pathogen.

In the present study, multivariable logistic regression analysis demonstrated that vector-related exposure was more influential than most host demographic factors in shaping the epidemiology of bovine TBD. Among the evaluated species, sex was significantly associated only with *B. bigemina* infection, with female cattle exhibiting higher odds of infection, whereas no significant sex-related associations were observed for *B. bovis*, *B. ovata*, or *T. orientalis*. Sex-related differences in susceptibility to TBP have been variably reported across endemic regions. For example, Rizk et al. (2017) reported higher odds of *B. bovis* infection in male cattle, attributing the pattern to differences in grazing behavior, work-related stress, and hormonal influences that may increase tick exposure [22]. Similar associations have been documented in water buffaloes from southern Thailand, where males exhibited significantly higher odds of *B. bovis* infection in multivariable models [23]. However, the absence of a significant sex effect for *B. bovis* in the present study suggests that such associations may be study-dependent and influenced by local management practices, host utilization, and vector ecology. Notably, the presence of ectoparasites emerged as a consistent and significant predictor of infection for *B. bigemina*, *B. ovata*, and *T. orientalis*, reinforcing the importance of tick vectors in protozoal transmission. Comparable findings have been reported from South Asia, where host-related factors such as age, sex, and breed influenced *T. annulata* infection risk, although the direction and magnitude of these associations varied by production system and ecological setting [24]. Furthermore, pregnancy, parturition, and lactation are commonly associated with production stress and endocrine fluctuations that can modulate immune function and may increase susceptibility to tick infestation and pathogen establishment. Field studies have reported higher tick infestation levels during lactation and other physiologically demanding periods, with proposed links to changes in hormones such as prolactin and progesterone and the associated immune modulation [25]. Taken together, the present findings suggest that vector exposure plays a central role in the epidemiology of bovine TBP infections, while host-related factors contribute to a species-specific and epidemiological setting.

In the present study, cattle infected with *Babesia* and *Theileria* spp. exhibited significantly lower PCV values compared with non-infected animals, indicating infection-associated anemia (*p* = 0.042). Although the reduction in PCV was moderate, it likely reflects early or subclinical hematological effects in naturally infected cattle. These findings are consistent with previous molecular epidemiological studies from Thailand, which reported significantly lower PCV values in infected cattle compared with uninfected cattle (*p* < 0.0001), with mean PCV values ranging from approximately 26.9% to 27.8% in infected animals [18]. Collectively, these results suggest that subclinical haemoprotozoan infections may compromise cattle health and productivity even in the absence of overt clinical signs.

Molecular analysis of tick samples in the present study confirmed the presence of *B. bovis* and *B. bigemina* DNA in ticks collected from cattle, supporting the role of local tick populations in pathogen transmission. Similar low-level detection of *Babesia* spp. in *R. microplus* has been reported previously, including a study by Jaimes et al. (2018) [26]. Comparable findings have been documented in regional surveys; for example, a large-scale molecular study from China in 2025 detected *B. bigemina* and *T. orientalis* among multiple tick species [27], while Habibi et al. (2021) identified *T. annulata* in tick samples even when host infection levels varied [28]. The absence of *B. ovata* and *T. orientalis* in tick samples in the present study, despite their detection in cattle blood, may reflect low parasite loads, vector specificity, or a mismatch between host parasitemia and tick feeding. Overall, these findings align with regional evidence that tick infection rates are often lower than host prevalence but remain epidemiologically important in maintaining transmission cycles.

The obtained *B. ovata* sequences showed comparatively lower nucleotide identity with available reference sequences in GenBank compared with other detected species. This reduced sequence identity may reflect underlying genetic variability within *B. ovata* populations or the limited number of well-characterized reference sequences currently available in public databases.

Another consideration is the potential impact of co-infections. Ticks frequently transmit multiple pathogens, and mixed infections involving piroplasms together with bacterial agents (e.g., *Anaplasma* spp. and other rickettsial organisms) have been documented in cattle and can worsen clinical outcomes, complicate diagnosis, and influence anemia severity [29] since the present study targeted protozoa only. Integrated surveillance, including bacterial TBP, would be valuable in future border area risk assessments.

Some limitations of this study should be acknowledged. First, the cross-sectional study design reflects infection status at a single point in time and therefore cannot account for seasonal variation in tick activity and protozoal transmission, which are known to influence TBD. Second, despite sensitive molecular methods being used, parasite burden was not quantified. This may partly explain why *B. ovata* and *T. orientalis* were detected in cattle blood but not in the corresponding tick samples. Third, environmental sampling was not performed, which may have underestimated the overall vector infection load in the study area. Despite these constraints, the combined analysis of cattle and tick samples provides valuable insight into the epidemiology of bovine tick-borne protozoa in this border region.

## 5. Conclusions

In conclusion, this study presents a complete molecular investigation of bovine TBP in cattle and associated ticks along the Thai-Myanmar border. The high overall prevalence of *Babesia* and *Theileria* spp. confirms that haemoprotozoan infections are endemic in this region. Among the detected species, *B. bigemina* was the most prevalent, followed by *B. bovis*, *T. orientalis*, and *B. ovata*. Species-specific differences in prevalence and risk factor associations highlight the complex interplay between host susceptibility, tick exposure, and local ecological conditions. The detection of multiple protozoan species in cross-border cattle suggests that animal movement may play an important role in maintaining and spreading these pathogens in western Thailand. This study fills critical gaps in the epidemiology of bovine TBP in Thailand and represents the first molecular characterization of infections in cross-border imported cattle along the Thai–Myanmar border. Furthermore, the significantly lower PCV values observed in infected cattle indicate that even subclinical infections can have measurable effects on cattle health and productivity. Overall, these findings highlight the need for strengthened molecular surveillance, effective tick control measures, and routine screening of imported cattle to reduce the risk of pathogen introduction and ongoing transmission in border areas.

## Figures and Tables

**Table 1:** Primer sequences, target genes, amplicon sizes, and optimized cycling conditions used in conventional and nested PCR assays for the molecular detection and differentiation of *Babesia* and *Theileria* species.

PCR Type	Target Organism	Target Gene	Primer Sequence with Direction (5′→3′)	Amplicon Size (bp)	Cycling Conditions	Reference
cPCR	*Babesia/Theileria* spp.	*18S rRNA*	F: GACACAGGGAGGTAGTGACAAG	403	95 °C–5 min; (×35) 94 °C–60 s, 60 °C–60 s, 72 °C–60 s; 72 °C–10 min	[12]
			R: CTAAGAATTTCACCTCTGACAGT			
	*Babesia ovata*	AMA-1	F: GATACGAGGCTGTCGGTAGC	504	95 °C–5 min; (×40) 95 °C–30 s, 56 °C–60 s, 72 °C–60 s; 72 °C–10 min	[13]
			R: AGTATAGGTGAGCATCAGTG			
	*Theileria orientalis*	MPSP	F: CTTTGCCTAGGATACTTCCT	776	94 °C–5 min; (×35) 94 °C–30 s, 58 °C–45 s, 72 °C–60 s; 72 °C–7 min	[14]
			R: ACGGCAAGTGGTGAGAACT			
nPCR	*Babesia bovis*	SBP-4	F1: AGTTGTTGGAGGAGGCTAAT	907 (outer), 503 (inner)	95 °C–5 min; (×35) 94 °C–60 s, 55 °C–60 s, 72 °C–60 s; 72 °C–10 min	[7]
			R1: TCCTTCTCGGCGTCCTTTTC			
			F2: GAAATCCCTGTTCCAGAG		95 °C–5 min; (×35) 94 °C–60 s, 55 °C–60 s, 72 °C–60 s; 72 °C–10 min	
			R2: TCGTTGATAACACTGCAA			
	*Babesia bigemina*	RAP-1a	F1: GAGTCTGCCAAATCCTTAC	879 (outer), 412 (inner)	95 °C–5 min; (×35) 94 °C–60 s, 55 °C–60 s, 72 °C–60 s; 72 °C–10 min	[7]
			R1: TCCTCTACAGCTGCTTCG			
			F2: AGCTTGCTTTCACAACTCGCC		95 °C–5 min; (×35) 94 °C–60 s, 55 °C–60 s, 72 °C–60 s; 72 °C–10 min	
			R2: TTGGTGCTTTGACCGACGACA			
	*Theileria parva*	p104	F1: ATTTAAGGAACCTGACGTGACTGC	496 (outer), 277 (inner)	95 °C–5 min; (×35) 94 °C–30 s, 65 °C–30 s, 72 °C–60 s; 72 °C–5 min	[15]
			R1: TAAGATGCCGACTATTAATGACACC			
			F2: GGCCAAGGTCTCCTTCAGATTACG		95 °C–5 min; (×35) 94 °C–30 s, 60 °C–30 s, 72 °C–60 s; 72 °C–5 min	
			R2: TGGGTGTGTTTCCTCGTCATCTGC			
	*Theileria annulata*	TAMS-1	F1: GTAACCTTTAAAAACGT	721 (outer), 453 (inner)	95 °C–5 min; (×30) 94 °C–60 s, 58 °C–60 s, 72 °C–60 s; 72 °C–10 min	[16]
			R1: GTTACGAACATGGGTTT			
			F2: CACCTCAACATACCCC		94 °C–5 min; (×35) 94 °C–30 s, 58 °C–45 s, 72 °C–60 s; 72 °C–7 min	
			R2: TGACCCACTTATCGTCC			

AMA: Apical membrane antigen; MPSP: Major piroplasm surface protein; SBP: Spherical body protein; RAP: Rhoptry-associated protein; TAMS: *Theileria annulata* merozoite surface.

**Table 2:** Prevalence and geographic distribution of tick-borne protozoa based on species detected in cattle from Kanchanaburi Province, Thailand. The chart illustrated the overall and relative infection of *Babesia bovis*, *B. bigemina*, *B. ovata*, and *Theileria orientalis* across three geographical areas and cross-border cattle.

District/Group	Total (N)	*Babesia*/*Theileria* spp. Positive (*n*)	Prevalence % (95% CI)	*B. bovis*, *n* (%)	*B. bigemina*, *n* (%)	*B. ovata*, *n* (%)	*T. orientalis*, *n* (%)
Thong Pha Phum	50	28	56.0 (42.2–69.8)	7 (14.0)	6 (12.0)	0 (0.0)	2 (4.0)
Sangkhlaburi	70	38	54.3 (42.6–65.9)	6 (8.6)	10 (14.3)	2 (2.9)	1 (1.4)
CBC group	38	15	39.5 (23.9–55.0)	0 (0.0)	1 (2.6)	5 (13.2)	6 (15.8)
Overall	158	81	51.3 (43.4–59.0)	13 (8.2)	17 (10.8)	7 (4.4)	9 (5.7)

**Table 3:** Overall and specific prevalence of tick-borne protozoa and multivariable logistic regression assessing associations between risk factors and infection status of *Babesia bovis*, *B. bigemina*, *B. ovata*, and *Theileria orientalis* detected in cattle (*n* = 158).

Factor	Subgroup (N)	Overall % (*n*; 95% CI)	*B. bovis* % (*n*; 95% CI)	OR	*p*-Values	*B. bigemina* % (*n*; 95% CI)	OR	*p*-Values	*B. ovata* % (*n*; 95% CI)	OR	*p*-Values	*T. orientalis* % (*n*; 95% CI)	OR	*p*-Values
Sex	Female (90)	50.00 (45; 39.88–60.12)	11.11 (10; 6.15–19.26)	0.6	0.485	7.78 (7; 3.82–15.19)	3.81	0.017 *	2.22 (2; 0.61–7.74)	0.39	0.407	2.22 (2; 0.61–7.74)	1.12	0.901
	Male (68)	52.94 (36; 41.24–64.33)	4.41 (3; 1.51–12.19)			14.71 (10; 8.19–25.00)			7.35 (5; 3.18–16.09)			8.82 (6; 4.11–17.94)		
Breed	Local (99)	46.46 (46; 36.96–56.24)	8.08 (8; 4.15–15.14)	0.77	0.669	9.09 (9; 4.86–16.38)	1.11	0.853	7.07 (7; 3.47–13.88)	—	Dropped	7.07 (7; 3.47–13.88)	0.94	0.948
	Crossbred (59)	59.32 (35; 46.59–70.91)	8.47 (5; 3.67–18.35)			13.56 (8; 7.03–24.54)			0.00 (0; 0.00–6.11)			3.39 (2; 0.93–11.54)		
Ectoparasite	Absent (51)	41.18 (21; 28.75–54.83)	1.96 (1; 0.35–10.30)	5.3	0.136	3.92 (2; 1.08–13.22)	7.03	0.023 *	11.76 (6; 5.50–23.38)	0.05	0.036 *	13.73 (7; 6.81–25.72)	0.13	0.024 *
	Present (107)	56.07 (60; 46.62–65.11)	11.21 (12; 6.53–18.59)			14.02 (15; 8.68–21.85)			0.93 (1; 0.17–5.11)			1.87 (2; 0.51–6.56)		
Age category	≤2 years (26)	46.15 (12; 28.76–64.54)	11.54 (3; 4.00–28.98)	0.49	0.198	11.54 (3; 4.00–28.98)	0.6	0.297	3.85 (1; 0.68–18.89)	0.71	0.635	3.85 (1; 0.68–18.89)	1.22	0.766
	2–5 years (114)	51.75 (59; 42.67–60.72)	12.28 (14; 7.46–19.56)			12.28 (14; 7.46–19.56)			5.26 (6; 2.43–11.01)			6.14 (7; 3.01–12.13)		
	>5 years (18)	55.56 (10; 33.72–75.44)	0.00 (0; 0.00–17.59)			0.00 (0; 0.00–17.59)			0.00 (0; 0.00–17.59)			5.56 (1; 0.99–25.76)		

* Statistically significant associations (*p* < 0.05) are indicated. OR and 95% CI derived from multivariable logistic regression. Overall prevalence represents the proportion of animals positive for at least one pathogen species.

**Table 4:** Comparison of packed cell volume (PCV) values, standard deviations (SD), confidence intervals (CI), and results of the independent samples *t*-test evaluating the hematological impact of protozoan infection.

*Babesia* or *Theileria* Infection	Total (N)	Mean ± SD	Std. Error	95% CI for Mean	*t* (df)	*p*-Value
Non-infected	77	36.21 ± 4.77	0.54	35.13–37.30	2.05 (156)	0.042
Infected	81	34.6 ± 5.08	0.56	33.48–35.73		

**Table 5:** Prevalence of *Babesia* and *Theileria* DNA in ticks collected from infested cattle across three study areas. The table summarizes the total number of tick-infested cattle, the number of PCR-positive tick samples, and the species.

Types of Protozoan DNA	Districts/Group	Total (N)	Positive (*n*)	Prevalence (%)	*p*-Value
*Babesia/Theileria* spp.	Thong Pha Phum	47	6	12.8	0.005 *
	Sangkhlaburi	46	4	8.7	
	CBC group	20	8	40	
*B. bovis*	Thong Pha Phum	47	3	6.4	0.39
	Sangkhlaburi	46	1	2.2	
	CBC group	20	2	10	
*B. bigemina*	Thong Pha Phum	47	0	0	0.48
	Sangkhlaburi	46	1	2.2	
	CBC group	20	0	0	

* Statistically significant associations (*p* < 0.05) are indicated.

**Table 6:** BLAST + 2.17.0 analysis of gene sequences of *Babesia* and *Theileria* species detected in blood and tick samples, showing the closest GenBank matches and percent sequence identity.

Sample Type	Species	Target Gene	GenBank Accession No.	Percent Identity (%)
Blood	*Babesia bovis*	SBP-4	AB772320	98.1
	*Babesia bigemina*	RAP-1a	MK481015	98.3
	*Babesia ovata*	AMA-1	AB703297	92.5
	*Theileria orientalis*	MPSP	LC438487	100
Tick	*Babesia bovis*	SBP-4	AB772320	98.1
	*Babesia bigemina*	RAP-1a	MK481015	98.8

## Data Availability

The original contributions presented in this study are included in the article. Further inquiries can be directed to the corresponding author.

## Abbreviations

The following abbreviations are used in this manuscript:

TBD	Tick-borne disease
TBP	Tick-borne protozoa
CBC	Cross-border cattle
PCV	Packed cell volume
